# Characterization and comparative profiling of piRNAs in serum biopsies of pediatric Wilms tumor patients

**DOI:** 10.1186/s12935-025-03780-4

**Published:** 2025-04-26

**Authors:** Fatma S. Mohamed, Deena Jalal, Youssef M. Fadel, Samir F. El-Mashtoly, Wael Z. Khaled, Ahmed A. Sayed, Mohamed A. Ghazy

**Affiliations:** 1 Biotechnology Program, Institute of Basic and Applied Science Egypt-Japan University of Science and Technology, New Borg El-Arab City, Alexandria Egypt; 2 Biochemistry ProgramFaculty of Science, Minia University, El-Minia, Egypt; 3 Genomics and Metagenomics Program, Department of Basic Research, Children’s Cancer Hospital Egypt, Cairo, 57357 Egypt; 4 Bioinformatics Group, Center for Informatics Science, School of Information Technology and Computer Science, Nile University, Giza, Egypt; 5 Leibniz Institute of Photonic Technology, Albert Einstein-Straße 9, 07745 Jena, Germany; 6 Department of Pediatric Oncology, National Cancer Institute, Cairo, Egypt; 7 Department of Pediatric Oncology, Children’s Cancer Hospital Egypt, Cairo, 57357 Egypt; 8 Consultant Pediatric Oncology, Mouwasat Hospital, Dammam, Saudi Arabia; 9 Department of Biochemistry, Faculty of Science, Ain Shams University, Cairo, Egypt

**Keywords:** PiRNAome profiling, Non-transposon-related PiRNAs, Non-germline functions, Diagnostic potential, Blood-based biomarkers, Wilms tumor

## Abstract

Piwi-interacting RNAs (piRNAs) are small non-coding RNAs involved in transposon silencing and linked to cancer progression. However, their role in Wilms tumors (WT) remains unexplored. We conducted a thorough analysis and characterization of piRNAs in serum liquid biopsies of WT patients. Our study examined their expression patterns and functional annotations related to WT pathogenesis, as well as their clinical potential for diagnosis and monitoring. We identified 307 piRNAs expressed in WT serum samples, with 4% classified as repeat-related and 96% as non-repeat-related. The most abundant repeat-related piRNAs originated from LINEs retrotransposon, while tRNA-derived piRNAs were the most prevalent among non-repeat-related piRNAs. Furthermore, a distinct profile of 34 piRNAs showed significant differential expression in WT patients compared to healthy controls—22 downregulated and 12 upregulated. The target genes of differentially expressed piRNAs exhibited significant enrichment in biological pathways related to cytokine activity, inflammatory responses, TGF-beta signaling, p38 MAPK, and ErbB signaling. These genes are also involved in DNA damage response, DNA methylation, cell cycle regulation, as well as kidney development and function. Low expression levels of several piRNAs, especially piR-hsa-1,913,711, piR-hsa-28,190, piR-hsa-28,849, piR-hsa-28,848, and piR-hsa-28,318, showed significant diagnostic potential as non-invasive biomarkers for WT (AUC > 0.8, *p* < 0.05). Their expression levels also significantly correlated with adverse pathological features, including metastasis, anaplasia, and bilateral WT development. In conclusion, non-transposon-related piRNAs may serve as reliable biomarkers for WT and possess potential non-germline functions, particularly in regulating DNA methylation, cell growth, immune responses, and immune responses. Further studies are warranted to elucidate their functional significance.

**Supplementary Information:**

The online version contains supplementary material available at 10.1186/s12935-025-03780-4.

## Graphical Abstract

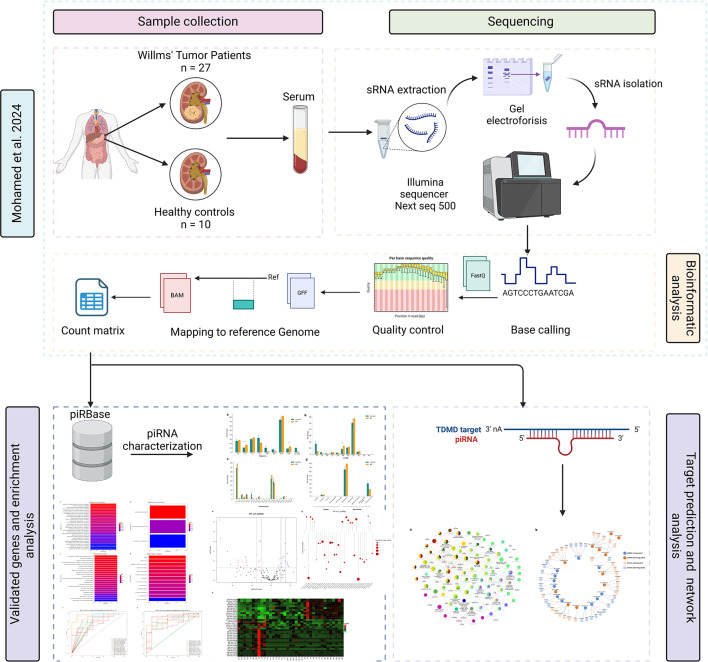

## Introduction

Malignant renal tumors account for 5% of pediatric cancers, accounting for 14,000 diagnoses and 5,000 deaths annually [1]. Wilms tumor (WT) is the most common pediatric renal tumor, accounting for more than 85% of renal tumors [2, 3]. It is typically diagnosed incidentally between ages 3 to 5 years [4]. WTs are embryonal renal tumors that arise from blastemal cells failing to differentiate and complete the mesenchymal-epithelial transition (MET) during early nephron development [5]. Most WT patients are typically treated with a combination of nephrectomy and chemotherapy, in addition to radiation therapy for high-risk patients. Recent clinical studies focus on improve risk stratification, intensify treatment for high-risk patients, and reduce the chemotherapeutic dosages for those with favorable prognoses [6]. Therefore, identifying blood-borne biomarkers is crucial for early disease detection, risk assessment, and development of novel therapeutic targets.

High-throughput sequencing techniques have identified regulatory RNAs, particularly small non-coding RNAs (sncRNAs) in various cancer types; these include microRNAs, piRNAs, siRNAs, and snoRNAs, which have been shown to play critical roles in regulating gene expression at both transcriptional and translational levels. They also contribute to cancer development and progression and hold promise as diagnostic and prognostic biomarkers [7].

PIWI-interacting RNAs (piRNAs), a newly identified class of small noncoding RNAs, are characterized by their specific length of 24–31 nucleotides and the presence of 2′-O-methyl modification at the 3′-end [8]. They were first discovered in germline cells, where they play a crucial role in transposon silencing and in maintaining genome stability [9]. They interact with a subfamily of Argonaute proteins, specifically the PIWI proteins, to form the piRNA-induced silencing complex (piRISC). This complex guides piRNAs to target complementary RNA molecules or transposon sequences, thereby suppressing their activity and safeguarding genomic integrity from transposon-induced abnormalities [10].

Although most information on piRNAs and their biogenesis has been primarily derived from studies conducted on *Drosophila* and *Caenorhabditis elegans* (*C. elegans*) [11, 12], these findings are also relevant to other species, including humans. The biogenesis of piRNAs occurs through two principal pathways: the primary processing pathway and the ping-pong cycle amplification. In the primary pathway, long single-stranded RNAs originating from piRNA clusters are processed into mature piRNAs, which typically feature uridine (U) at their 5′ end—a characteristic known as the 1U bias. This preference for uridine at the first position of 5′ uracil-containing piRNAs is believed to enhance their ability to effectively target transposons for silencing. The endonuclease Zucchini plays a crucial role in forming both ends of these piRNAs. The ping-pong cycle, on the other hand, amplifies piRNAs through a feedback mechanism involving the proteins *Aubergine (Aub)* and *AGO3*, which facilitate the cleavage of transposon or target transcripts to generate secondary piRNAs. Notably, these secondary piRNAs frequently exhibit a 10 A bias, which complements the primary piRNAs in this process [13].

PiRNAs primarily function in germline processes, such as silencing transposable elements and regulating gene expression during gametogenesis [9]. However, recent studies have revealed that they also play broader roles in somatic cells, including transcription suppression, translation modulation, mRNA stability regulation, stress response management, and protein interactions [13, 14].

Furthermore, piRNAs have emerged as significant players in the development and progression of various human cancers, including gastric, hepatocellular, renal cell, pancreatic, bladder, and breast carcinomas [15–19]. Dysregulation of piRNA expression has been shown to affect gene expression and promote tumor growth, highlighting their potential as valuable diagnostic and therapeutic targets in oncology [20]. Many piRNAs are implicated in key cancer processes such as proliferation, apoptosis, and metastasis, and may serve as potential biomarkers [15, 20]. For example, piRNA-823 promotes angiogenesis in multiple myeloma [21], while piR-8041 induces apoptosis in glioblastoma [22]. Additionally, piR-55,490 inhibits lung carcinoma growth via the mTOR pathway [23], and piR-651 promotes apoptosis in non-small cell lung cancer (NSCLC) [24]. In breast cancer, piR-21,285 modulates tumor development through epigenetic mechanisms [25], with upregulated piR-4987, piR-20,365, piR-20,485, and piR-20,582 serving as potential biomarkers [26].

Moreover, piRNAs have been linked to negative clinicopathological features in various cancers, including clinical stage, metastasis, and relapse [15, 27]. Specifically, high levels of piR-823 correlate with the TNM stage in the serum of renal cell carcinoma [28]. Increased expression levels of piR-32,051, piR-39,894, and piR-43,607 are strongly associated with metastasis, advanced clinical stage, and poor survival in ccRCC [29]. Similarly, piR-4987 is also associated with tumor metastasis in breast cancer [26]. Due to their small size and stability, piRNAs can pass through the cell membrane and be detected in human body fluids such as blood or urine, making them promising non-invasive biomarkers for various cancers [30]. For instance, serum levels of piR-54,265 may serve as a valuable biomarker for the early detection and clinical monitoring of colorectal cancer. Its levels significantly decrease following surgery, but exhibit a notable increase upon tumor recurrence [31].

In this context, our previous research, which focused on profiling abnormal expression of miRNAs in serum biopsies of WT patients [32], identified a significant proportion of sequences annotated as piRNAs alongside miRNAs. Consequently, the present study aims to analyze expression signatures of piRNAs in WT patients compared to healthy controls, assessing their potential as non-invasive biomarker for diagnosing or monitoring this pediatric renal tumor. Additionally, we investigated the differential expression of piRNAs across various pathological subtypes of WT and performed functional annotation of this class of small noncoding RNAs to elucidate their roles in biological processes beyond germline functions. This study represents the first evidence of the involvement of piRNAs in WT pathogenesis, paving the way for further exploration into the regulatory functions of piRNAs in WT development.

## Materials and methods

### Study design and data source

This study builds upon our previously published analysis of small non-coding RNAs in WT patients [32]. The patient cohort, sample collection, RNA extraction, and sequencing procedures were conducted as described in our prior work [32], and no additional sample collection or sequencing was performed for this study. Briefly, serum samples were obtained from 37 children (10 healthy controls, 14 with favorable histology [FH-WT], and 13 with unfavorable histology [UnFH-WT]) from the Biorepository at Children Cancer Hospital Egypt (CCHE), 57,357. RNA was extracted from these samples using the miRNeasy Mini Kit (Qiagen), and small RNA sequencing libraries were prepared using the NEBNext Multiplex Small RNA Library Prep Set for Illumina. Sequencing was performed on the Illumina MiSeq platform, as detailed in our prior publication [32].

### NGS data processing and piRNA annotation

For this study, we utilized previously processed sequencing data from our prior work [32]. Briefly, raw sequencing reads underwent quality control using FastQC [33] and adapter trimming with Cutadapt [34]. High-quality reads were aligned to the GRCh38 reference genome using Bowtie 2 [35]. Within this database, miRBase served as a reference for miRNAs, piRBase for piRNAs, and GeneCards for other small RNAs. The resulting BAM files containing sncRNAs were then quantified for each sample using FeatureCounts [36], with the following parameters: --minOverlap 15, --fracOverlap 0.95, and --fracOverlapFeature 0.95 resulting in full small RNA count tables. Differential expression analysis of small ncRNAs between WT patients and healthy controls was conducted using the R/Bioconductor DESeq2 package [37], with significance determined by an absolute log2 fold change (log2FC ≥ 1 or ≤-1) and an adjusted p-value ≤ 0.05.

**Fig. 1:**
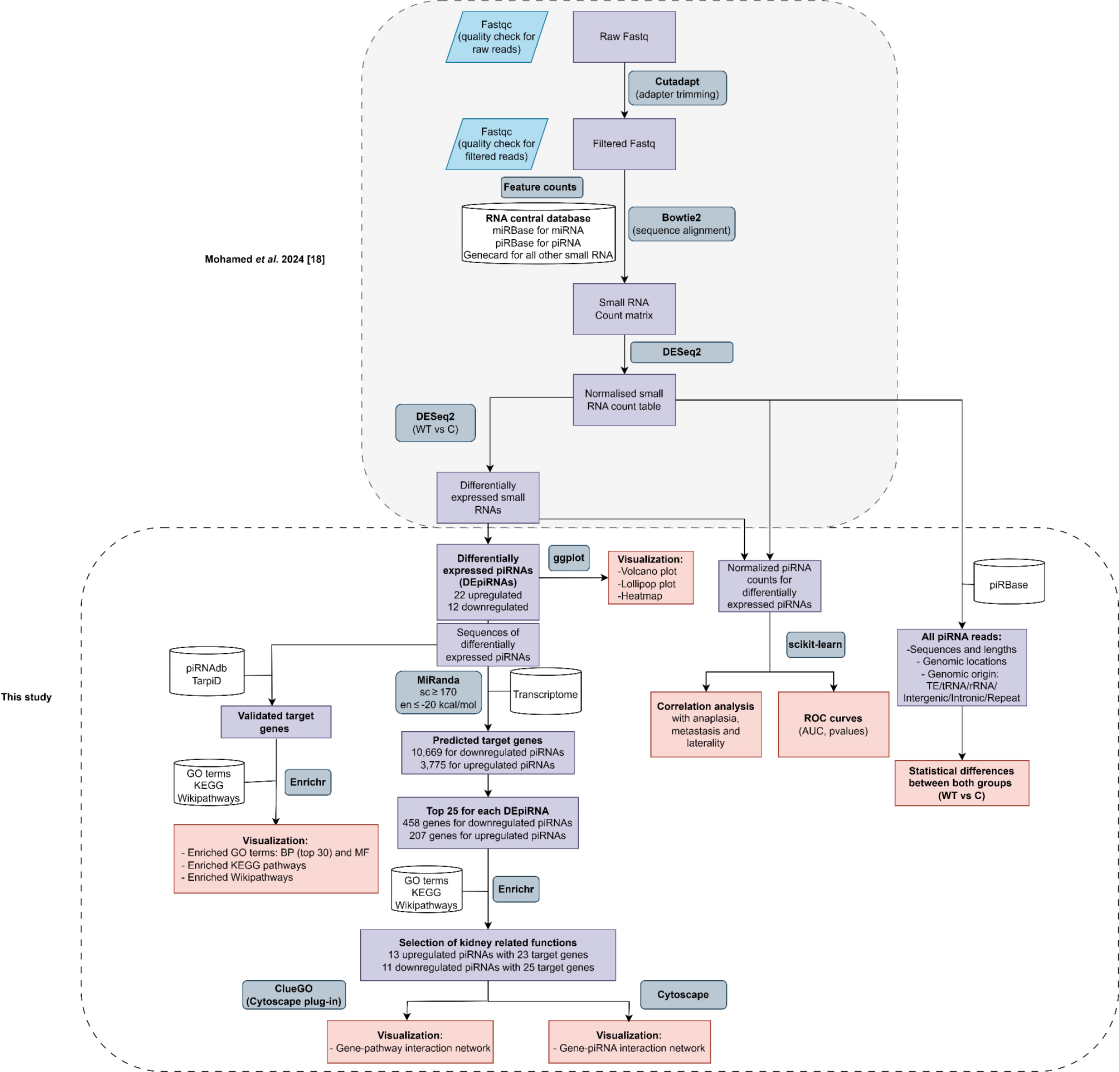
Workflow of the piRNA sequencing analysis pipeline. It includes the following steps: Raw Data Processing, Read Alignment, Count Matrix Generation, piRNA characterization, Differential Expression Analysis, target prediction, Functional Enrichment Analysis, and Statistical Analysis. This diagram was designed using Draw.io online diagram software

In the current study, we specifically focused on piRNA analysis, utilizing previously generated and normalized small RNA count tables. Differential expression results from our original dataset were used to identify piRNAs differentially expressed in WT vs. healthy controls and between WT subtypes. The raw sequencing data has been deposited in the NCBI BioProject database under accession ID PRJNA1112148. A schematic representation of the piRNA characterization and expression analysis workflow is provided in Figure. 1.

### Retrieval of piRNA sequences and identification of their genomic origins

The piRNA sequences were retrieved from the RNA Central database [38], and the nucleotide bias at the first and tenth positions in piRNA sequences was assessed. The chromosomal locations of the identified set of piRNAs were examined in relation to regions of transposon repeats, pseudogenes, non-coding RNAs (lncRNAs, miRNAs, tRNAs, rRNAs, and others), as well as introns and exons (CDS, 3’/5’ UTR), to identify the genomic origins of the candidate piRNAs using the piRBase [39] and piRNAquest V.2 [40] databases. piRNAs were considered repeat related if they originated from transposable elements (TE, SINE and LTR), transposons or simple repeats, and otherwise considered non-repeat related.

### Differential expression analysis of Circulating piRNA in Wilms tumor

The visualization of the DEpiRNA was accomplished using the ggplot2 R package [41] for volcano and lollipop plots, while the complex Heatmap R package [42] was employed to generate a heatmap of piRNA expression levels across all samples.

### Identification of validated and predicted target genes of differentially expressed piRNA in Wilms tumor

The databases TarpiD [43] and piRNAdb [44], which comprise putative and validated piRNA targets, were used to identify the experimentally validated target genes of DEpiRNA in WT patients. Their nucleotide sequences were used to avoid naming discrepancies.

The miRanda software [45], a widely used algorithm for predicting miRNA**-**mRNA interactions, was used to identify potential targets of DEpiRNA in WT. This was done by analyzing sequence complementarity between piRNA and mRNA transcripts obtained from GENCODE Release 46 (https://www.gencodegenes.org/human/release_46.html). The current study employed a rigorous selection process to identify the most promising piRNA targets based on their alignment score (sc ≥ 170) and binding energy (en ≤ -20.0 kcal mol^− 1^), ensuring that only the most relevant targets were included in the functional analysis. The piRNA-target duplexes identified through miRanda predictions underwent additional filtration based on the level of sequence complementarity within the primary (2–11 nts) and secondary seed site (12–21 nts). The filtration criteria required that the primary seed site exhibit a minimum of 7 consecutive Watson-Crick base pairs, while the secondary seed site contain at least 6 consecutive Watson-Crick base pairs.

### Functional enrichment analysis of differentially expressed piRNA in Wilms tumor

The Enrichr platform [46], a comprehensive gene set enrichment analysis web server, was used to perform functional and pathway enrichment analyses of target genes of DEpiRNA in WT. For enrichment analyses, we employed the Gene Ontology (GO) [47], Kyoto Encyclopedia of Genes and Genomes (KEGG) pathway [48], and WikiPathways [49] libraries using the enrichR package in R /Bioconductor. The results were subsequently visualized using the ggplot2 R package [41]. Gene sets exhibiting nominal p-values of < 0.05 and q values of < 0.25 were deemed statistically significant. Enriched terms were then filtered and ranked based on a p-value threshold of < 0.05. The interaction network of DEpiRNA and their target genes was created using Cytoscape software version 3.10.3 [50], incorporating the CluePedia plugin version 1.5.10 [51] for pathway insights based on integrated experimental and in silico data.

### Statistical analysis

Receiver operating characteristic (ROC) curve analysis [52] was performed to assess the diagnostic efficacy of the DEpiRNA using Python scikit-learn library [53]. The area under the ROC curve (AUC) value provided a quantitative measure of piRNA’ performance in distinguishing between groups. Additionally, the Spearman’s rank correlation coefficient test [54] was used to analyze the relationship between piRNA expression and WT clinicopathological characteristics.

## Results

### Abundance of Circulating piRNA in the serum of Wilms tumor patients

In our previous analysis of WT-miRNome [32], we found that approximately 14% of total sequenced reads were annotated as small non-coding RNAs. The most abundant class of scnRNA reads in both WT serum samples and control subjects was piRNA, which accounted for 77.7% (3,589,379 reads) of the aligned reads, followed by miRNAs at 20.6% (951,982 reads). Each sample obtained an average of 97,010 reads for piRNA, ranging from 6,958 to 369,956 reads. After analyzing reads mapped to the piRBase database, we identified a comprehensive catalog of 307 piRNA that are commonly expressed in WT and control serum samples. The fraction distribution of reads mapped to different ncRNAs was previously published [32] and shown in Figure S1.

### Exploring the PiRNA landscape in serum of Wilms tumor

**Fig. 2:**
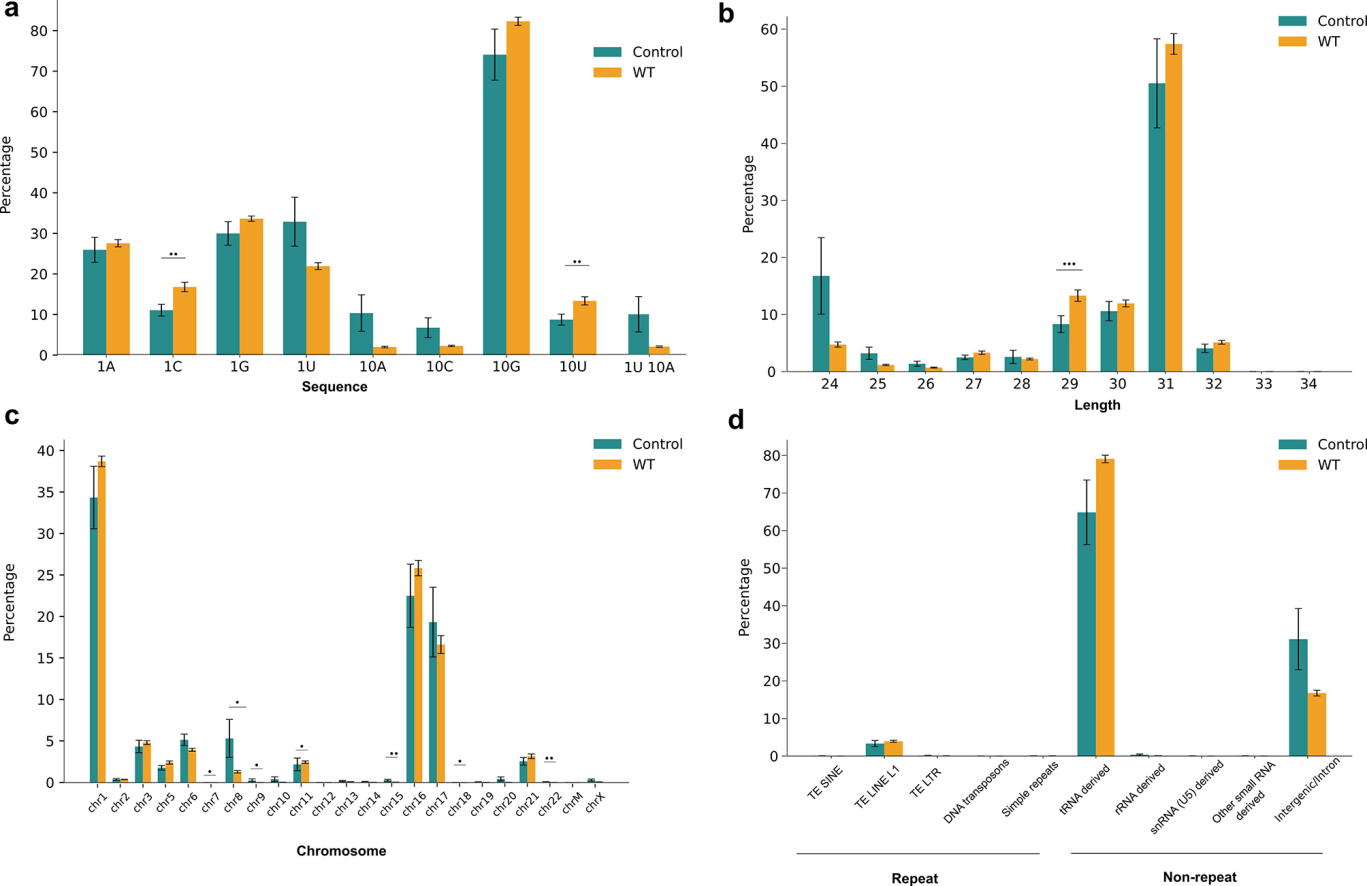
Characterization of circulating piRNA in WT serum samples. (**a**) Nucleotide bias at the 1st and 10th positions of piRNA sequences. (**b**) Length distribution of piRNA reads in WT and healthy controls. (**c**) Chromosomal distribution of piRNA expression across all human chromosomes. (**d**) Categorization of identified piRNA into repeat-related and non-repeat-related piRNA, including their origins from various transposon elements and translation machinery molecules. Statistical significance was assessed using the Mann-Whitney U test, with significant p-values (*p* < 0.05) indicated in the figure

Our examination of the nucleotide bias at the first and tenth positions in piRNA sequences revealed that piRNA of WT patients did not show a strong preference for uridine (U) at the 1st position and adenine (A) at the 10th position (Fig. 2A and Table S2). However, there was a significant increase in the proportion of cytosine (C) at the first position, and Uracil (U) at the 10th position within the WT-piRNA compared to those of healthy controls. In contrast, the proportions of bases A and G at the first position did not show significant differences between the two groups.

By analyzing the length distribution of piRNA reads in WT serum samples, we observed that the putative piRNA displayed a length range of 24–34 nucleotides (nts), with the highest frequency at 31 nts. Notably, the proportion of piRNA reads with 29 nts in length showed a significant increase in WT samples compared to healthy controls (Fig. 2B and Table S3).

### Genomic diversity of PiRNA origins in Wilms tumor

The piRNA have been found to be derived from both genic and non-genic loci across all human chromosomes. The analysis of piRNA expression across all chromosomes indicated a predominant expression pattern of piRNA on chromosomes 1, 6, 11, 16 and 17 in all samples (Fig. 2C), suggesting that these chromosomes serve as the primary loci for piRNA expression.

Furthermore, the identified piRNA were categorized as “repeat-related piRNA” if they originated from transposable elements (SINEs, TEs or LTRs), transposons, or simple repeats, and “non-repeat-related piRNA” if they originated from other genomic regions. Each WT sample had an average of 2986.54 (4%) reads for repeat-related piRNA and 83956.78 (96%) reads for non-repeat-related piRNA) Fig. 2D). Our findings revealed that repeat-derived piRNA originate from different types of Transposon elements (TE), including Long interspersed nuclear elements (LINE), Short interspersed nuclear elements (SINE), Long terminal repeat elements (LTR), DNA repeat elements and simple repeats (micro-satellites). Among these, the retrotransposon LINE-derived piRNA were the most abundant category, showing the highest frequency of repeat-related piRNA reads. In addition to repeat-related piRNA, our study found that approximately 16.7% of WT-piRNA reads originate from non-genic loci, including intronic and intergenic regions, and about 79.2% of piRNA reads originate from translation machinery molecules, including tRNAs, rRNAs, and snRNAs (U5) (Fig. 2D and Table S4). Among these, tRNA-derived piRNA reads are the most prevalent in WT patients and healthy controls, highlighting their potential role in regulating WT gene expression. This observation suggests a potential link between the dysregulation of translation machinery and WT progression.

### Dysregulation of PiRNA expression in the circulation of Wilms tumor patients

**Fig. 3:**
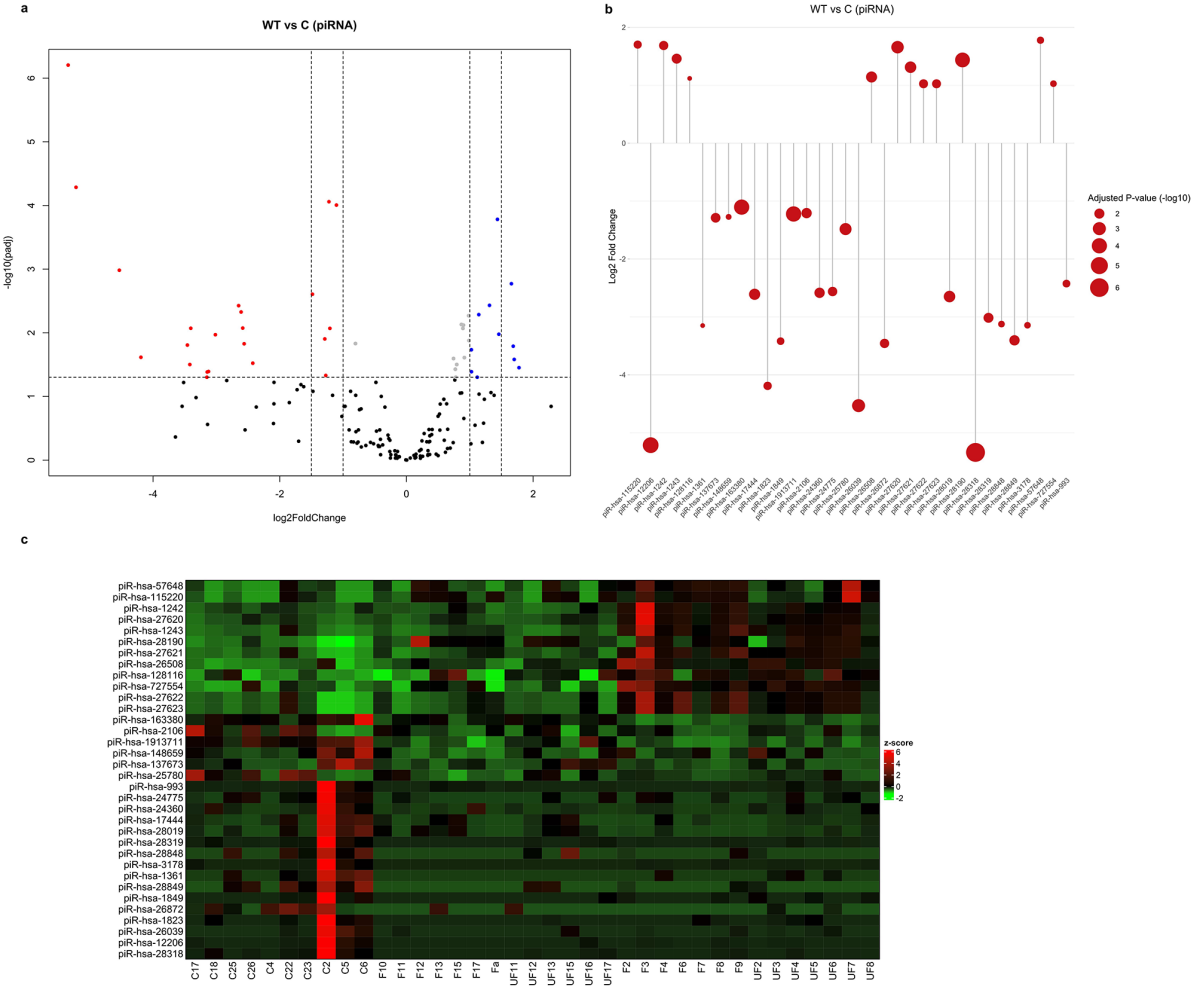
Differential expression analysis of piRNA in WT serum samples. **(a)** Volcano plot displays all expressed piRNA in serum samples, illustrating the log2 fold change (FC) against the adjusted p-value (-log10). In this plot, red dots represent downregulated piRNA, while blue dots indicate upregulated piRNA. **(b)** Lollipop plot visualizing individual DEpiRNA that display significant variations according to log2 fold change and adjusted p-values. The size of the red circles corresponds to the level of significance, as indicated by the adjusted p-values. **(c)** Heatmap displays the expression levels of piRNA in WT and control samples. Labels denote “F” for favorable WTs, “UF” for unfavorable WTs, and “C” for healthy controls. Red color represents upregulated piRNA, while blue signifies downregulated ones. Visualization of the DEpiRNA was performed using the ggplot2 R package for volcano plots and lollipop plots, and the complex Heatmap R package for heatmap

Our research identified a distinct expression profile of 34 piRNA that showed significant differential expressions in the serum samples of WT patients compared to those of healthy individuals, with 12 piRNA upregulated and 22 piRNA downregulated. The log2 fold change of downregulated piRNA varied from − 1.105147303 to -5.34046587, with the most substantial downregulation observed in piR-hsa-28,318 and piR-hsa-12,206, which originate from chromosome 9 and 20, respectively. Conversely, the log2 fold change of upregulated piRNA ranged from 1.024895846 to 1.77670514, with the most significant upregulation seen in piR-hsa-57,648 and piR-hsa-115,220, which originate from chromosome 6 and 1, respectively. The fold changes of all expressed piRNA and their statistical significance in WT were depicted in a volcano plot (Fig. 3A) and summarized in Table 1. The individual piRNA that showed significant differential expression in WT samples were visualized in a lollipop plot (Fig. 3B). A heatmap representing the z-scores of DEpiRNA (Fig. 3C) illustrates significant expression differences between WT and control serum samples.

**Table 1:** Differential expression profiles of piRNA in the serum of WT patients compared to healthy controls

piRBase Id	log2FoldChange	*p*-value	Adjusted *p*-value
piR-hsa-28,318	-5.34046587	3.27E-09	6.24E-07
piR-hsa-12,206	-5.215886646	5.42E-07	5.18E-05
piR-hsa-26,039	-4.532419859	5.18E-05	0.001041
piR-hsa-1823	-4.19153802	0.004378	0.02424
piR-hsa-26,872	-3.458270472	0.00238	0.015589
piR-hsa-1849	-3.419378179	0.006588	0.031491
piR-hsa-28,849	-3.404696639	0.000889	0.008487
piR-hsa-1361	-3.150200426	0.012918	0.04988
piR-hsa-3178	-3.144981842	0.009941	0.041277
piR-hsa-28,848	-3.124585935	0.009553	0.040546
piR-hsa-28,319	-3.016459647	0.001276	0.010719
piR-hsa-28,019	-2.649275192	0.000255	0.003747
piR-hsa-17,444	-2.610043613	0.000345	0.00471
piR-hsa-24,360	-2.584572467	0.000861	0.008429
piR-hsa-24,775	-2.561868282	0.002219	0.014873
piR-hsa-993	-2.425464371	0.00613	0.03002
piR-hsa-25,780	-1.484140016	0.000149	0.002478
piR-hsa-137,673	-1.288976322	0.001602	0.012488
piR-hsa-148,659	-1.273711987	0.011758	0.046786
piR-hsa-1,913,711	-1.223420061	1.5E-06	8.73E-05
piR-hsa-2106	-1.20836314	0.000927	0.00852
piR-hsa-163,380	-1.105147303	2.32E-06	9.86E-05
piR-hsa-27,622	1.024895846	0.003141	0.01846
piR-hsa-27,623	1.024895846	0.003141	0.01846
piR-hsa-727,554	1.026404197	0.009736	0.040869
piR-hsa-128,116	1.117549048	0.013188	0.04988
piR-hsa-26,508	1.141890501	0.000393	0.005182
piR-hsa-27,621	1.310886452	0.000242	0.003705
piR-hsa-28,190	1.436338019	4.39E-06	0.000165
piR-hsa-1243	1.458615974	0.001212	0.010524
piR-hsa-27,620	1.656706986	9.32E-05	0.001695
piR-hsa-1242	1.685662678	0.00255	0.016238
piR-hsa-115,220	1.701160868	0.005079	0.026217
piR-hsa-57,648	1.77670514	0.007834	0.035295

The analysis of piRNA expression in serum samples across WT histopathological subtypes showed no significant differences between WTs with favorable histology (anaplastic WTs) and those with unfavorable histology (non-anaplastic WTs). However, there was a marked difference in the number of exclusively expressed piRNA in each histopathological type. A total of 22 piRNA were found to be abnormally expressed only in non-anaplastic WTs, and 2 exhibited dysregulations solely in anaplastic WTs. Additionally, 12 piRNA were commonly expressed across both histopathological types of WT (Figure S2).

### Enrichment of differentially expressed piRNA in cellular processes and pathways associated with Wilms tumor pathogenesis and kidney function

**Fig. 4:**
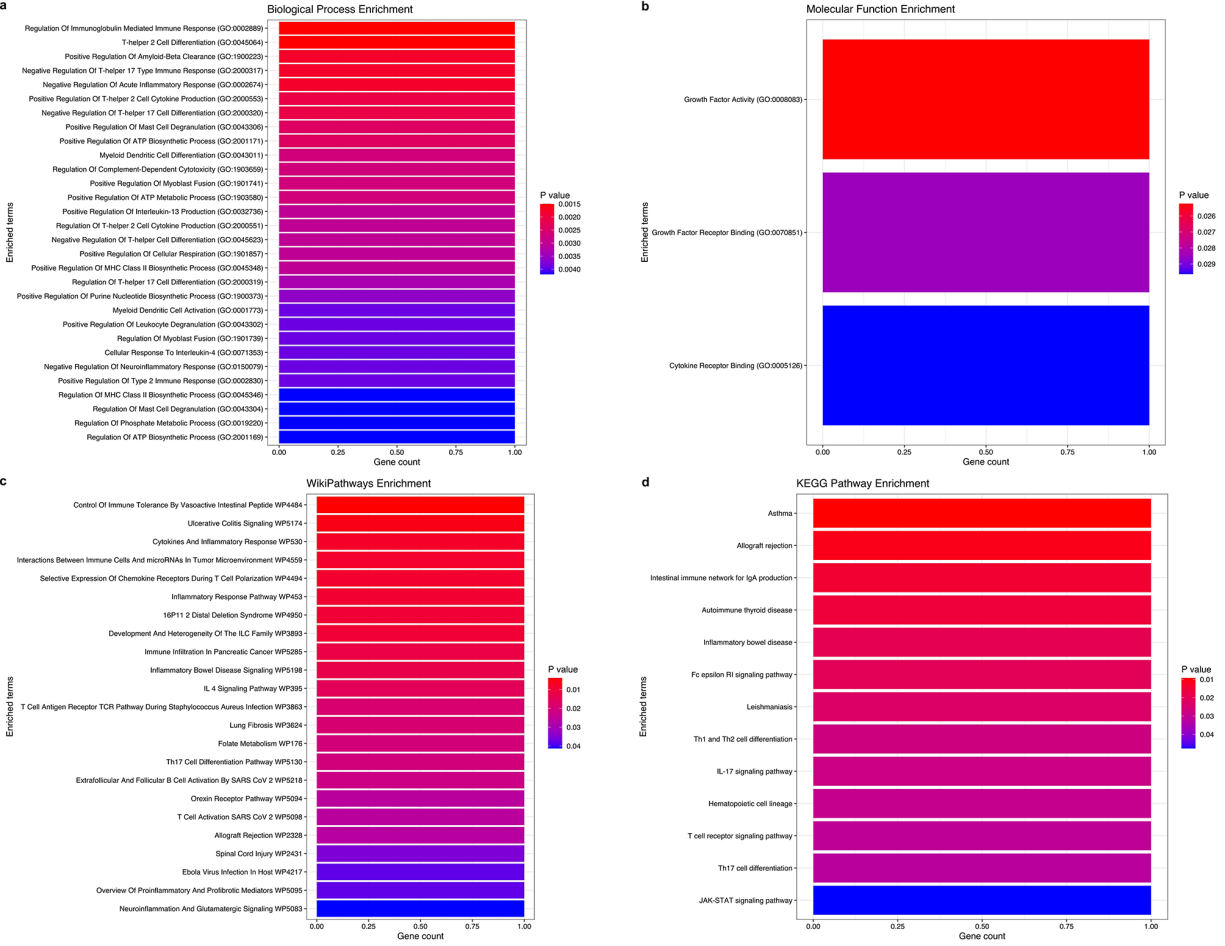
Functional enrichment analysis of validated target genes of DEpiRNA in WT. **(a**,** b)** The top GO terms (Biological processes and Molecular functions) of the six validated target genes linked to four DEpiRNA: piR-hsa-993, piR-hsa-26039, piR-hsa-24360, and piR-hsa-28318) in WT. **(c**,** d)** The top KEGG and WikiPathways related to these six validated target genes influenced by DEpiRNA in WT. The top enriched terms for the input gene set are ranked based on their p-values. The GO, KEGG, and WikiPathways analyses were conducted using the enrichR package and visualized using the ggplot2 R package

We used the TarpiD and piRNAdb databases to identify the validated target genes of DEpiRNA in WT. In the TarpiD database, piR-hsa-993 targets the *IL4* gene. According to the piRNAdb database, piR-hsa-26,039 targets *GCFC2*, piR-hsa-24,360 targets both *CXorf21* and *ERG28*, and piR-hsa-28,318 targets both *LINC01431* and *SEZ6L*. These genes are significantly enriched in immune response processes (Fig. 4A), including immunoglobulin regulation (GO:0002889), T-helper 2 cell differentiation (GO:0045064), and acute inflammatory response (GO:0002674). They are also involved in molecular functions (Fig. 4B) related to Growth Factor Activity (GO:0008083) and Cytokine Activity (GO:0005125). In line with this, the KEGG and WikiPathways analyses highlight their enrichment in pathways related to cytokines and Inflammatory Response (WP530) and IL19 Signaling (WP5422) (Fig. 4C, D).

Furthermore, we used the miRanda algorithm to predict potential target genes for all DEpiRNA in WT. The results indicated that the 22 downregulated piRNA were predicted to target a total of 10,669 genes, while the 12 upregulated piRNA were predicted to target a total of 3,775 genes. For functional enrichment analysis, we selected the top 25 target genes with the highest alignment score of ≥ 170 and the lowest binding energy of ≤ -20.0 kcal mol^− 1^, resulting in a selection of 458 target genes for downregulated piRNA and 207 target genes for upregulated piRNA.

The GO analysis (Table S5) indicated that the predicted target genes are significantly enriched in biological processes related to kidney development, such as ureteric bud morphogenesis and branching (GO:0060675, GO:0001658), as well as cellular processes related to DNA methylation (GO:0044030), endothelial cell proliferation (GO:0001938), and the regulation of reactive oxygen species metabolic processes (GO:2000378). The WikiPathway and KEGG analyses (Table S6) indicated that these predicted target genes of DEpiRNA are enriched in critical signaling pathways, such as *TGF-beta* signaling, *p38 MAPK*, *ErbB* signaling, and focal adhesions, which are involved in WT pathogenesis. In addition to these major pathways these targets showed significant enrichment in pathways related to specific renal conditions and functions (e.g., collecting duct acid secretion, mineral absorption, nephrotic syndrome, and the mesenchymal-epithelial transition (MET) in type 1 papillary renal cell carcinoma). Furthermore, they are also involved in immune response pathways (*IL-5*,* IL-3*,* IL-2* signaling pathways, and B cell receptor signaling) and in controlling G1/S cell cycle, DNA damage response, and infectious diseases.

**Fig. 5:**
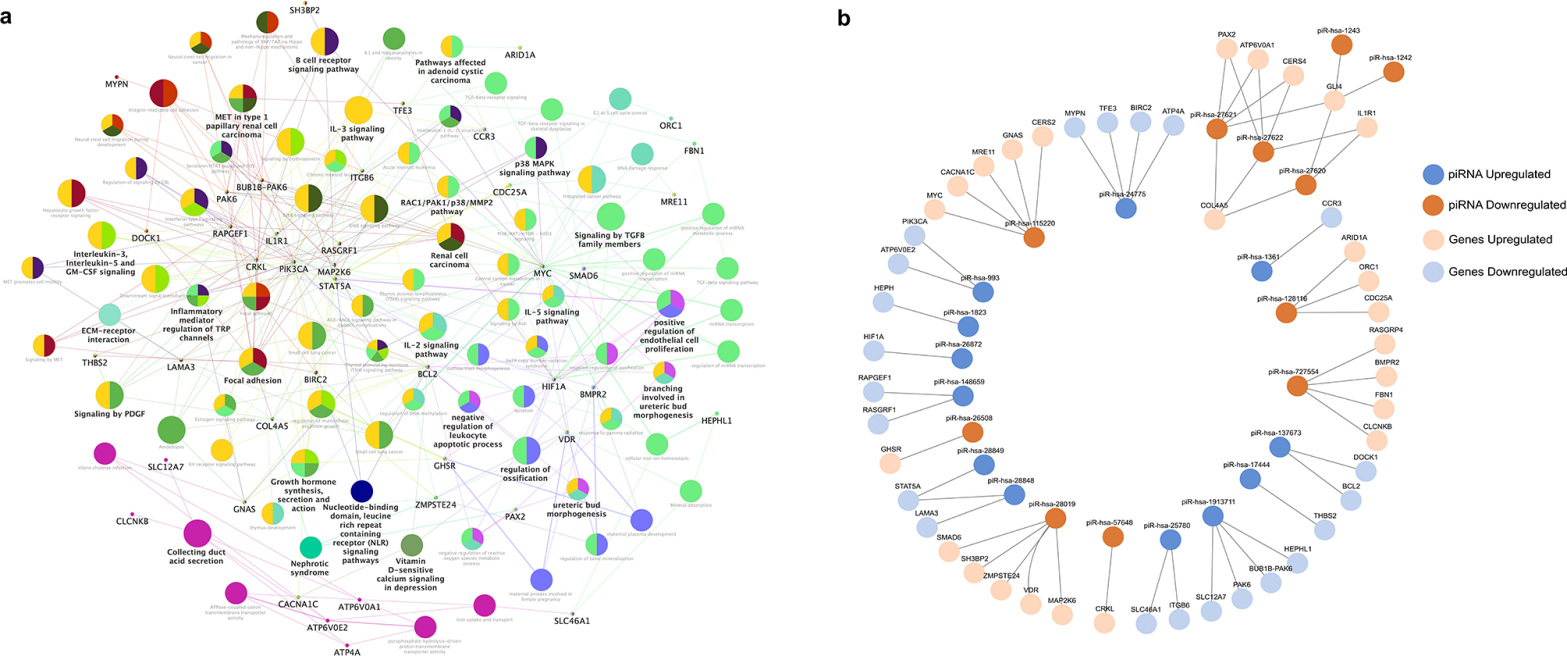
Interaction network of DEpiRNA and their predicted target genes in WT. **(a)** Network of the DEpiRNA target genes and their enrichment in various GO biological processes, KEGG, and WikiPathways. In this network, small circles represent the target genes of piRNA, while large colored circles indicate the most highly enriched terms of biological processes, KEGG pathways, and WikiPathways mediated by the piRNA targets. This network was constructed using Cytoscape software, incorporating the CluePedia plugin for pathway insights based on integrated experimental and in silico data. **(b)** Interaction network of DEpiRNA and their potential targets, as identified by the miRanda algorithm and visualized in Cytoscape. Upregulated piRNA are shown in blue and their targets in light blue, while downregulated piRNA are in orange with corresponding targets in light orange

We further filtered GO biological processes, KEGG pathways, and WikiPathways associated with kidney development and carcinogenesis. Our findings revealed that a total of 13 upregulated piRNA and their associated 23 target genes, along with 11 downregulated piRNA and their corresponding 25 target genes, are involved in these kidney-related functions. The relationships between these pathways and their associated genes are illustrated in Fig. 5A, while the interactions between these genes and DEpiRNA are depicted in Fig. 5B.

These networks revealed the regulatory roles of several piRNA in critical genes, including *MYC*,* BCL2*,* STAT5A*,* PAX2*,* IL1R1*,* COL4A5* and *MAP2K6*, which are key components of cancer-related pathways, such as *TGF-beta* signaling, *p38 MAPK*,* ErbB* signaling, focal adhesions, and other pathways associated with DNA damage and immune responses.

### Correlations between PiRNA expression levels and pathological characteristics of Wilms tumor

The Spearman rank correlation test was employed to evaluate the association between piRNA expression in WT serum samples and clinicopathological features of WT. Low levels of piR-hsa-28,190 were significantly associated with the presence of initial lung metastasis in WT patients (*r* = -0.3933, *p* = 0.042344). Similarly, decreased expression of piR-hsa-1,913,711 showed a significant association with an increased incidence of anaplasia (*r* = 0.4378, *p* = 0.022388). Reduced levels of piR-hsa-28,849 (*r* = 0.5826, *p* = 0.0014), piR-hsa-28,848 (*r* = 0.5826, *p* = 0.0014), and piR-hsa-28,318 (*r* = 0.3908, *p* = 0.0439) were strongly correlated with the development of bilateral WTs. No significant links were identified between piRNA expression and other clinicopathological factors, such as tumor stage, patient age, or gender. The detailed correlations between piRNA expression and clinicopathological features are summarized in Table S7.

### Circulating dysregulated piRNA as potential non-invasive biomarkers for Wilms tumor detection

**Fig. 6:**
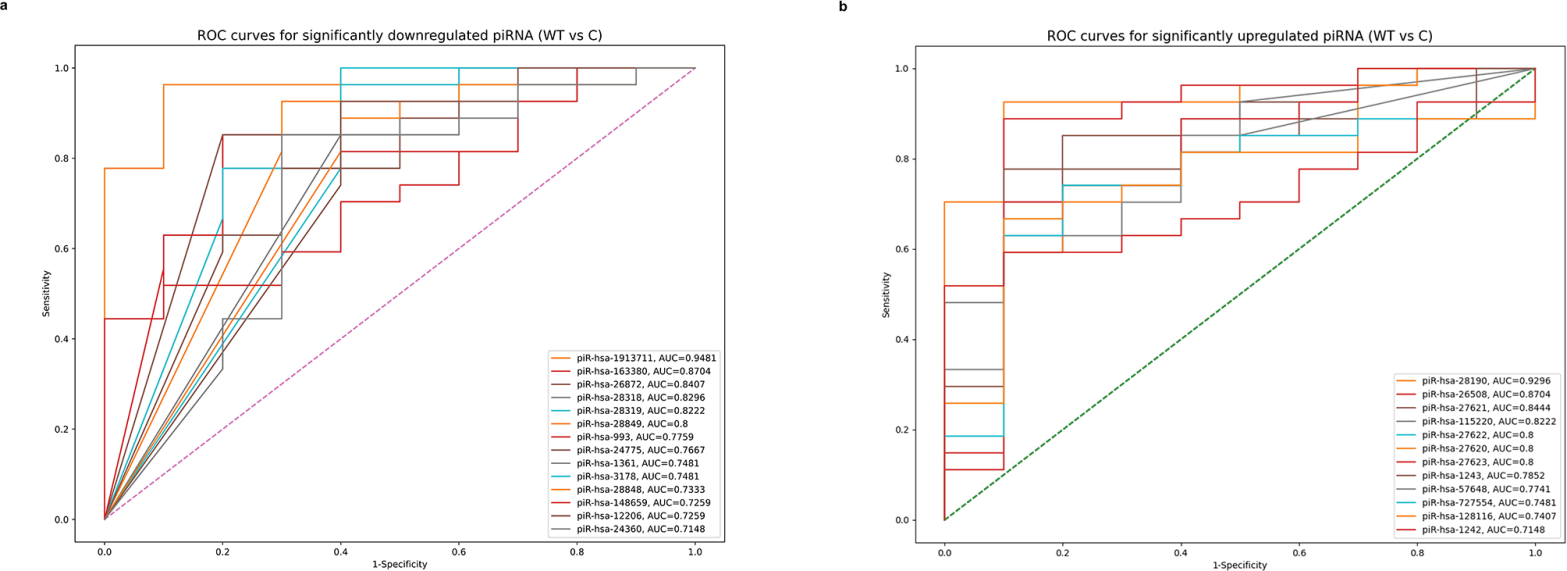
ROC curve analysis of piRNA as potential non-invasive biomarkers for WT. **(a)** ROC curves for significantly downregulated piRNA in WT, with the highest AUC values. **(b)** ROC curves for significantly upregulated piRNA, with the highest AUC values for detecting WT. The vertical axis represents sensitivity, while the horizontal axis represents 1 - specificity. The central diagonal line represents the reference (no discrimination), while the colored lines represent the ROC curves for downregulated and upregulated piRNA. The ROC curves were visualized using the Python scikit-learn library. The AUC values ranged from 0.7148 to 0.9481 (95% CI: 0.95), indicating good discriminatory ability

Based on the results of the ROC curve analysis, we found that 14 downregulated piRNA and 12 upregulated piRNA exhibited high specificity and sensitivity for distinguishing WT, with AUC values ranging from 0.7148 to 0.9481 and *p* < 0.05. Among the upregulated piRNA, piR-hsa-28,190 exhibited the highest AUC value of 0.9296, with a p-value of 0.0000781. In contrast, among the downregulated piRNA, piR-hsa-1,913,711 showed the highest AUC value of 0.9481, with a p-value of 0.0000377. These findings suggest that these piRNA may function as prospective biomarkers for WT detection due to their robust discriminatory ability. The ROC curves for significantly dysregulated piRNA with the highest AUC values are shown in Fig. 6A and B and summarized in Table S8.

## Discussion

In our previous study [32] on investigating miRNA expression patterns in WT serum samples, we found that over 77% of small RNA reads were identified as piRNA. Consequently, this abundance of piRNA in WT samples prompted us to conduct a thorough bioinformatics analysis and characterization of the WT-piRNome to clarify their role in WT pathogenesis and evaluate their potential as biomarkers for diagnosis and targeted therapy. This study is the first to investigate piRNA dysregulation in the serum of WT patients, suggesting the regulatory role of piRNA in WT pathogenesis.

piRNA are generally characterized by a 1U bias, and recent studies in Drosophila have clarified the mechanisms behind this phenomenon [55]. This bias arises through a two-step gating process: initially, 1 A and 1G piRNA are excluded during piRNA biogenesis, and subsequently, 1 C piRNA are filtered out during piRISC formation due to interactions with Piwi’s stem loop. In the serum of healthy controls, the expected predominance of 1U piRNA is observed, but this pattern is disrupted in WT patients, where a significant increase in 1 C piRNA is detected, with no significant changes in 1G or 1 A piRNA. This finding suggests a broader issue with PIWI loading and the formation of functional piRISC complexes. In contrast to the 1U bias, the 10 A bias was not observed in either WT patients or controls, which aligns with the absence of the ping-pong cycle during secondary piRNA biogenesis, typically seen in germline cells. Additionally, WT patients exhibited a slightly larger piRNA pool in their serum, further suggesting defects in piRNA biogenesis and PIWI loading.

Our analysis showed a predominant expression pattern of WT-piRNA on chromosomes 1, 6, 11, 16, and 17 across all samples, suggesting these chromosomes may be primary loci for expression. These findings are consistent with a previous study [56], where the top 100 piRNA in a cellular model of kidney development were mapped to chromosomes 1, 2, 11, and 17 across all cell lines examined. Similar patterns were observed in neuroblastoma cell lines, where the highest number of piRNA were found on chromosomes 1, 2, 6, 11, and 17 [57].

piRNA were initially believed to primarily function in the regulation of transposable elements and to be mainly derived from these regions within clusters, particularly in germline cells [58, 59]. However, recent research has revealed a more complex picture regarding the origins and functions of piRNA. In somatic cells, piRNA are found to frequently originate from both genic and non-genic regions across all human chromosomes. In neuroblastoma, only 17.8% of piRNA originate from transposable elements; however, approximately 18% are derived from protein-coding genes (5’ UTR, CDS, 3’ UTR), and 24% have intronic origins, while 15% are associated with transfer RNAs (tRNAs) [57]. In epithelial ovarian cancers, over 50% of piRNA are derived from various PCGs regions including both, and nearly one-third of piRNA are originate from other non-coding RNAs such as, tRNAs, lncRNAs, rRNAs, and srpRNAs). In contrast, the smallest proportion of piRNA comes from repeats or pseudogenes when compared to their overall genomic origins [60]. Consistent with these results, serum samples from our study also exhibit similar findings. Only 4% of all detected piRNA in serum samples of WT patients and healthy controls are derived from various types of transposable elements, 16.7% from intronic sequences and intergenic regions, and 79.2% from translation machinery molecules (such as tRNA, rRNA and snRNAs, etc.,). This diversity in piRNA origins indicates their involvement in complex regulatory roles, including gene expression, protein synthesis, translation, and the maintenance of genomic stability, extending beyond their initially proposed function in transposon silencing.

Our findings also highlight that most piRNA are derived from tRNAs, consistent with prior observations in both germline and somatic tumor cells. For instance, previous study demonstrated that defects in tRNA processing can induce replication stress and disrupt piRNA transcription in mutant ovaries, underscoring the connections between piRNA, tRNA processing, epigenetic regulation, and transposable element control [61]. Similarly, in tumor cells, HIWI2-associated piRNA in MDA-MB-231 breast cancer cells were shown to primarily originate from tRNAs, rather than retrotransposons or hypermethylated CpG islands [62].

Furthermore, the expression of piRNA have been found to be altered in various malignancies such as breast, prostate, and renal cell carcinomas [19, 26, 29, 63]. In renal cancer, deep sequencing profiling of piRNA showed that 19 piRNA were differentially expressed in benign kidney tissues compared to those in clear cell renal cell carcinoma, while 46 piRNA were linked to metastasis, highlighting their potential to distinguish tumor subtypes and indicate disease progression [29]. In the context of WT, our research uncovered a distinct piRNA expression profile comprising 34 piRNA with significant differential expression in serum samples of WT patients compared to healthy controls, with 22 downregulated and 12 upregulated piRNA.

Our functional enrichment analysis of the validated target genes (*IL4*,* GCFC2*,* CXorf21*,* ERG28*,* LINC01431*,* SEZ6L*) associated with four DEpiRNA (piR-hsa-993, piR-hsa-26039, piR-hsa-24360, and piR-hsa-28318) revealed significant enrichment in essential biological processes and vital pathways related to cytokine activity and inflammatory responses. Several previous studies emphasized the presence of highly immunosuppressive and inflammatory microenvironments in WT by detecting various inflammatory immune cells and inflammatory protein markers, particularly localized in the stroma of WT [64, 65]. These findings highlight the intricate interplay between these piRNA targets and the immune landscape of WT.

Furthermore, the GO and pathway analyses revealed that the predicted target genes of DEpiRNA are involved in vital kidney functions, such as renal absorption and morphogenesis and branching of ureteric buds. Recent research has indicated that both metanephric blastema cells and ureteric bud tip cells are involved in the development of nephrogenic rests and WT [66]. Previous study found that expression level of some piRNA (piRNA-42775, piRNA-36743 and piRNA-43108) were associated with proteinuria and renal function [67]. DNA methylation defects are commonly observed epigenetic anomalies in most WT cases [68]. The enrichment of the DEpiRNA targets in regulating DNA methylation indicates their potential role in mediating epigenetic modifications in WT.

The WikiPathway and KEGG analyses indicated that these predicted target genes of DEpiRNA are enriched in critical signaling pathways, such as TGF-beta signaling, p38 MAPK, ErbB signaling, and Focal adhesions, which are involved in WT pathogenesis. TGF-β1 was found to promote epithelial–mesenchymal transition (EMT), which enhances the migration and invasive properties of tumor cells in vivo [69]. Its overexpression was linked to tumor invasion and WT progression [70]. The p38 was found to be highly expressed and activated in the embryonic kidneys of rats, where it plays a crucial role in kidney development and nephrogenesis [71]. Numerous studies revealed that alterations in the ERBB signaling pathway are closely associated with the progression of various cancers and promote the transformation of tumor cells [72]. Additionally, a prior study indicated that *CCT4* may play a role in WT development by activating the ERBB signaling pathway [73]. Focal adhesion kinase (FAK) was found to be implicated in the progression of various pediatric renal tumors [74] and its inhibition led to decreased cell survival, proliferation, migration, and invasion in the primary WT model [75]. Collectively, all these findings suggest that piRNA may play a critical role not only in transposon silencing but also in regulating various cellular processes and pathways involved in WT pathogenesis.

In addition, piRNA have been found to be associated with adverse tumor characteristics, including clinical stage and metastasis, across various cancers [26, 29]. In this study, the findings of the Spearman rank correlation test indicated a significant relationship between piRNA expression levels and adverse pathological features of WT, such as the incidence of anaplasia, tumor metastasis, and bilateral WT tumors. Reduced expression of piR-hsa-28,190 was associated with WT metastasis, while lower expression levels of piR-hsa-1,913,711 were correlated with the occurrence of anaplasia. Additionally, decreased expression of piR-hsa-28,849, piR-hsa-28,848, and piR-hsa-28,318 was correlated with the development of bilateral WT tumors. These results suggest that reduced expression of these piRNA may be indicative of WT progression and disease severity.

Serum piRNA have emerged as promising biomarkers for the detection of various malignancies, including gastrointestinal, lung, and colorectal cancers [64–67]. In the present study, several DEpiRNA exhibited high specificity and sensitivity for WT, highlighting their potential as non-invasive diagnostic biomarkers. Notably, piR-hsa-28,190 and piR-hsa-1,913,711 demonstrated the highest diagnostic performance, with AUC values exceeding 0.9, underscoring their strong potential as minimally invasive and highly reliable biomarkers for WT diagnosis.

To further validate these findings, future studies should focus on large-scale, multicenter clinical trials to assess the reproducibility and reliability of these biomarkers across diverse patient cohorts. Additionally, functional studies are needed to elucidate the molecular mechanisms underlying their association with WT progression. Implementing piRNA-based detection in clinical practice could significantly enhance early WT detection and improve patient outcomes through more personalized and targeted therapeutic strategies.

## Conclusion

Our thorough analysis of the WT-piRNome has revealed a distinct piRNA expression profile in the serum samples of WT patients compared to healthy controls, with many piRNA showing promise as reliable non-invasive biomarkers for WT detection and progression. Beyond their established roles in genome stability and transposon silencing, these piRNA may influence key cellular processes and pathways involved in WT pathogenesis. However, their expression in WT tissue biopsies has not yet been investigated. Therefore, further validation of these non-transposon-related piRNA in a larger cohort of WT serum and tissue samples is needed to evaluate their efficacy as clinical biomarkers. Furthermore, exploring their regulatory function may offer valuable insights into WT pathogenesis.

## Electronic supplementary material

Below is the link to the electronic supplementary material.

Supplementary Material 1: Table S1. Clinicopathological characteristics of WT patients and heathy individuals [33]. Table S2. The base frequency of piRNA sequences at the 1st and 10th positions. Table S3. The length distribution of all expressed piRNA in WT. Table S4. Comparative analysis of piRNA read distribution originating from diverse genomic sources in WT and healthy control samples. Table S5. The Gene Ontology-Biological Processes enrichment of the predicted target genes of DEpiRNA in WT. Table S6. The KEGG Pathways and WikiPathways Enrichment of the predicted target genes of DEpiRNA in WT. Table S7. The correlation between Serum piRNA Expressions and Clinicopathological Characteristics of Wilms Tumor Patients. Table S8. The ROC analysis of DEpiRNA in WT compared to healthy controls. Figure S1. Percentages of aligned reads to different small ncRNAs in both WT and healthy control samples in our previously published study [33]. Figure S2. A venn diagram of the common and unique dysregulated piRNA between Favorable and Unfavorable histology WTs. The diagram was visualized using Venny tool v 2.1.

## Data Availability

The RNA-seq dataset supporting the findings of the current study is openly available in NCBI’s BioProject database under accession number [PRJNA1112148] https://www.ncbi.nlm.nih.gov/bioproject/?term=PRJNA1112148.The dataset used in this study builds on our earlier analysis of small non-coding RNAs (sncRNAs) in Wilms tumor patients at 10.3389/fmolb.2024.1453562. The small RNA-Seq analysis pipeline for (both piRNA and miRNAs) can be accessed at https://github.com/you27-mohamed/Small-RNAseq-analysis.

## Abbreviations

WT: Wilms’ Tumor
MET: Mesenchymal-Epithelial Transition
sncRNAs: small non-coding RNAs
piRNAs: PIWI-interacting RNAs
piRISC: piRNA Silencing Complex
C. elegans: Caenorhabditis Elegans
Aub: Aubergine (Aub)
FH-WT: Favorable Histology Wilms tumor
UnFH-WT: Unfavorable Histology Wilms tumor
DEpiRNAs: Differentially Expressed piRNAs
GO: Gene Ontology
KEGG: Kyoto Encyclopedia of Genes and Genomes pathway
ROC: Receiver Operating Characteristic
AUC: Area Under the ROC Curve
TE: Transposon Elements
LINE: Long Interspersed Nuclear Elements
SINE: Short Interspersed Nuclear Elements
LTR: Long Terminal Repeat Elements
IL4: Interleukin 4
GCFC2: GC-rich Sequence DNA-Binding Factor2
ERG28: Ergosterol Biosynthesis 28 Homolog
LINC01431: long Intergenic Non-Protein Coding RNA 1431
SEZ6L: Seizure Related 6 Homolog Like
TGF-β: Transforming Growth Factor Beta
p38 MAPK: p38 Mitogen-Activated Protein Kinases
STAT5A: Signal Transducer and Activator of Transcription 5 A
Pax-2: Paired box gene 2
IL1R1: Interleukin 1 Receptor Type 1
COL4A5: Collagen Type IV Alpha 5 Chain
MAP2K6: Mitogen-Activated Protein Kinase Kinase 6
FAK: Focal Adhesion Kinase
TNM: Tumor-Node-Metastasis
CCHE: Children’s Cancer Hospital Egypt
