# Supplementary material for: Characterization and comparative profiling of piRNAs in serum biopsies of pediatric Wilms tumor patients
Source: Cancer Cell Int. 2025 Apr 26;25:163. doi: 10.1186/s12935-025-03780-4 (PMC12034122; doi:10.1186/s12935-025-03780-4)
Supplement: Supplementary file 1 — Supplementary Material 1: Table S1. Clinicopathological characteristics of WT patients and heathy individuals [33]. Table S2. The base frequency of piRNA sequences at the 1st and 10th positions. Table S3. The length distribution of all expressed piRNA in WT. Table S4. Comparative analysis of piRNA read distribution originating from diverse genomic sources in WT and healthy control samples. Table S5. The Gene Ontology-Biological Processes enrichment of the predicted target genes of DEpiRNA in WT. Table S6. The KEGG Pathways and WikiPathways Enrichment of the predicted target genes of DEpiRNA in WT. Table S7. The correlation between Serum piRNA Expressions and Clinicopathological Characteristics of Wilms Tumor Patients. Table S8. The ROC analysis of DEpiRNA in WT compared to healthy controls. Figure S1. Percentages of aligned reads to different small ncRNAs in both WT and healthy control samples in our previously published study [33]. Figure S2. A venn diagram of the common and unique dysregulated piRNA between Favorable and Unfavorable histology WTs. The diagram was visualized using Venny tool v 2.1. [file 12935_2025_3780_MOESM1_ESM.docx]

Supplementary Material

Fatma S. Mohamed, Deena Jalal, Youssef M. Fadel, Samir F. El-Mashtoly, Wael Z. Khaled, Ahmed A. Sayed*, Mohamed A Ghazy

*** Correspondence:** Ahmed A. Sayed [Ahmed.Sayed@57357.org](mailto:Ahmed.Sayed@57357.org)

**Table S1.** Clinicopathological characteristics of WT patients and heathy individuals [33].

| Characteristics | Wilms Tumor cases  (n=27) | Healthy controls cases  (n=10) |
| --- | --- | --- |
| Diagnostic Age /  Age at sample collection | 1-8 Years | 1.5 - 6 years |
| Gender  Female  Male | 14 (51.8%)  13 (48%) | 6(60%)  4(40%) |
| Tumor Histopathology  Non-anaplastic WTs (Favorable histology FH-WT)   - FH-WT-Mixed type - FH-WT-Stromal - FH-WT-Epithelial   Anaplastic WTs (Unfavorable histology UnFH-WT)   - Diffuse Anaplasia - Focal Anaplasia | 14 (51.8%)  7 (25.9%)  5 (18.5%)  2 (7%)  13 (48%)  10 (37%)  3 (11%) |  |
| Clinical Stage  II  III  IV  V | 1 (3.7%)  15 (55.5%)  7 (25.9%)  4 (14.8%) |  |
| Laterality  Unilateral Tumors  Bilateral Tumors | 23 (85%)  4 (14.8%) |  |
| Initial Metastasis to lung  Present  Absent | 9 (33%)  18(66.6%) |  |
| Congenital Anomalies | Absent | Absent |
| Viral infection , diabetes, other kidney diseases | Absent | Absent |

**Table S2.** The base frequency of piRNA sequences at the 1st and 10th positions.

| **Nucleotide Bias**  **of piRNAs** | **Average percentage of piRNA counts in controls (%)** | **Average percentage of piRNA counts in WT (%)** | **p-value** |
| --- | --- | --- | --- |
| 1A | 25.98 | 27.59 | 0.79756 |
| 1C | 11.08 | 16.80 | 0.00726 |
| 1G | 30.02 | 33.64 | 0.71952 |
| 1U | 32.90 | 21.94 | 0.31303 |
| 10A | 10.37 | 2.00 | 0.07258 |
| 10C | 6.77 | 2.28 | 0.17673 |
| 10G | 74.09 | 82.32 | 0.85080 |
| 10U | 8.76 | 13.39 | 0.00385 |
| 1U 10A | 9.87 | 1.89 | 0.00433 |

**Table S3.** The length distribution of all expressed piRNAs in WT.

| **piRNA length** | **Average percentage of piRNA count in controls (%)** | **Average percentage of piRNA count in WT (%)** | **p-value** |
| --- | --- | --- | --- |
| 24 nts | 16.77 | 4.75 | 0.13684 |
| 25 nts | 3.23 | 1.17 | 0.07258 |
| 26 nts | 1.37 | 0.72 | 0.12804 |
| 27 nts | 2.50 | 3.32 | 0.07819 |
| 28 nts | 2.57 | 2.21 | 0.09718 |
| 29 nts | 8.34 | 13.32 | 0.00429 |
| 30 nts | 10.60 | 11.95 | 0.13683 |
| 31 nts | 50.51 | 57.40 | 0.90472 |
| 32 nts | 4.09 | 5.13 | 0.08415 |
| 33 nts | 0.01 | 0.01 | 0.04118 |
| 34 nts | 0.06 | 0.01 | 0.07163 |

**Table S4.** Comparative analysis of piRNA read distribution across genomic sources in Wilms tumor and healthy controls

|  | **piRNA Genomic Origins** | **Average percentage of piRNA count in controls (%)** | **Average percentage of piRNA count in WT (%)** | **p-value** |
| --- | --- | --- | --- | --- |
| **Repeat–related piRNAs** | SINE | 0.05 | 0.01 | 0.53732 |
|  | LINE L1 | 3.34 | 3.92 | 0.05332 |
|  | LTR | 0.11 | 0.05 | 0.95908 |
|  | DNA transposon | 0.02 | 0.004 | 0.11606 |
|  | Simple repeat | 0.05 | 0.06 | 0.85080 |
| **Non-repeat related piRNAs** | tRNA-derived piRNAs | 64.85 | 79.06 | 0.77128 |
|  | rRNA-derived piRNAs | 0.36 | 0.11 | 1 |
|  | snRNA-derived piRNAs | 0.03 | 0.0004 | 0.47197 |
|  | Other small RNAs | 0.06 | 0.02 | 0.71952 |
|  | Other origins of piRNAs  (Introns and Intergenic regions) | 31.12 | 16.76 | 0.46216 |

**Table S5**. Gene Ontology-Biological Processes enrichment analysis of predicted targets of differentially expressed piRNAs in WT.

| **Ontology ID** | **Gene Ontology-Biological processes Enrichment** | **P-Value Corrected with Bonferroni** | **Associated Genes** |
| --- | --- | --- | --- |
| GO:0001658 | branching involved in ureteric bud morphogenesis | 0.00281 | [BCL2, MYC, PAX2] |
| GO:0001893 | maternal placenta development | 0.00203 | [BMPR2, GHSR, VDR] |
| GO:0001938 | positive regulation of endothelial cell proliferation | 0.00029 | [BMPR2, CCR3, GHSR, HIF1A, STAT5A] |
| GO:0003151 | outflow tract morphogenesis | 0.00152 | [BMPR2, CRKL, HIF1A, SMAD6] |
| GO:0006879 | cellular iron ion homeostasis | 0.00075 | [HEPHL1, HIF1A, MYC, SLC46A1] |
| GO:0007595 | Lactation | 0.00342 | [HIF1A, STAT5A, VDR] |
| GO:0009678 | pyrophosphate hydrolysis-driven proton transmembrane transporter activity | 0.00143 | [ATP4A, ATP6V0A1, ATP6V0E2] |
| GO:0010332 | response to gamma radiation | 0.00343 | [BCL2, MYC, ZMPSTE24] |
| GO:0019829 | ATPase-coupled cation transmembrane transporter activity | 0.00192 | [ATP4A, ATP6V0A1, ATP6V0E2] |
| GO:0030278 | regulation of ossification | 0.00003 | [BCL2, BMPR2, HIF1A, SMAD6, VDR, ZMPSTE24] |
| GO:0030279 | negative regulation of ossification | 0.00273 | [BCL2, HIF1A, SMAD6] |
| GO:0030500 | regulation of bone mineralization | 0.00161 | [BMPR2, HIF1A, VDR, ZMPSTE24] |
| GO:0040014 | regulation of multicellular organism growth | 0.00000 | [BCL2, GHSR, GNAS, PIK3CA, STAT5A, ZMPSTE24] |
| GO:0044030 | regulation of DNA methylation | 0.00143 | [MYC, PIK3CA, ZMPSTE24] |
| GO:0048538 | thymus development | 0.00361 | [BCL2, CRKL, ZMPSTE24] |
| GO:0060135 | maternal process involved in female pregnancy | 0.00192 | [BMPR2, GHSR, VDR] |
| GO:0060675 | ureteric bud morphogenesis | 0.00079 | [BCL2, MYC, PAX2] |
| GO:0061614 | miRNA transcription | 0.00235 | [HIF1A, MYC, SMAD6] |
| GO:1902893 | regulation of miRNA transcription | 0.00235 | [HIF1A, MYC, SMAD6] |
| GO:1902895 | positive regulation of miRNA transcription | 0.00349 | [HIF1A, MYC, SMAD6] |
| GO:2000107 | negative regulation of leukocyte apoptotic process | 0.00346 | [BCL2, GHSR, HIF1A] |
| GO:2000378 | negative regulation of reactive oxygen species metabolic process | 0.00353 | [BCL2, HIF1A, PAX2] |
| GO:2000630 | positive regulation of miRNA metabolic process | 0.00328 | [HIF1A, MYC, SMAD6] |

**Table S6**. KEGG and WikiPathways enrichment analysis of predicted target genes of differentially expressed piRNAs in WT.

| **Pathway ID** | **KEGG Pathways Enrichment** | **P-Value Corrected with Bonferroni** | **Associated Genes** |
| --- | --- | --- | --- |
| KEGG:04012 | ErbB signaling pathway | 0.00000 | [BUB1B-PAK6, CRKL, MYC, PAK6, PIK3CA, STAT5A] |
| KEGG:04350 | TGF-beta signaling pathway | 0.00203 | [BMPR2, FBN1, MYC, SMAD6] |
| KEGG:04510 | Focal adhesion | 0.00000 | [BCL2, BIRC2, BUB1B-PAK6, COL4A5, CRKL, DOCK1, ITGB6, LAMA3, PAK6, PIK3CA, RAPGEF1, RASGRF1, THBS2] |
| KEGG:04512 | ECM-receptor interaction | 0.00169 | [COL4A5, ITGB6, LAMA3, THBS2] |
| KEGG:04750 | Inflammatory mediator regulation of TRP channels | 0.00220 | [GNAS, IL1R1, MAP2K6, PIK3CA] |
| KEGG:04933 | AGE-RAGE signaling pathway in diabetic complications | 0.00227 | [BCL2, COL4A5, PIK3CA, STAT5A] |
| KEGG:04935 | Growth hormone synthesis, secretion and action | 0.00000 | [CACNA1C, CRKL, GHSR, GNAS, MAP2K6, PIK3CA, STAT5A] |
| KEGG:04966 | Collecting duct acid secretion | 0.00000 | [ATP4A, ATP6V0A1, ATP6V0E2, CLCNKB, SLC12A7] |
| KEGG:04978 | Mineral absorption | 0.00340 | [HEPHL1, SLC46A1, VDR] |
| KEGG:05110 | Vibrio cholerae infection | 0.00346 | [ATP6V0A1, ATP6V0E2, GNAS] |
| KEGG:05146 | Amoebiasis | 0.00020 | [COL4A5, GNAS, IL1R1, LAMA3, PIK3CA] |
| KEGG:05211 | Renal cell carcinoma | 0.00000 | [BUB1B-PAK6, CRKL, HIF1A, PAK6, PIK3CA, RAPGEF1, TFE3] |
| KEGG:05220 | Chronic myeloid leukemia | 0.00109 | [CRKL, MYC, PIK3CA, STAT5A] |
| KEGG:05221 | Acute myeloid leukemia | 0.00235 | [MYC, PIK3CA, STAT5A] |
| KEGG:05222 | Small cell lung cancer | 0.00000 | [BCL2, BIRC2, COL4A5, LAMA3, MYC, PIK3CA] |
| KEGG:05230 | Central carbon metabolism in cancer | 0.00134 | [HIF1A, MYC, PIK3CA] |
| **Pathway ID** | **WikiPathways Enrichment** | **P-Value Corrected with Bonferroni** | **Associated Genes** |
| WP:127 | IL-5 signaling pathway | 0.00266 | [BCL2, MYC, STAT5A] |
| WP:185 | Integrin-mediated cell adhesion | 0.00000 | [BUB1B-PAK6, DOCK1, ITGB6, MAP2K6, MYPN, PAK6, RAPGEF1] |
| WP:1971 | Integrated cancer pathway | 0.00019 | [BCL2, CDC25A, MRE11, MYC] |
| WP:2032 | Thyroid stimulating hormone (TSH) signaling pathway | 0.00077 | [GNAS, MAP2K6, MYC, PIK3CA] |
| WP:2203 | Thymic stromal lymphopoietin (TSLP) signaling pathway | 0.00350 | [MYC, PIK3CA, STAT5A] |
| WP:23 | B cell receptor signaling pathway | 0.00017 | [CRKL, MAP2K6, MYC, RAPGEF1, SH3BP2] |
| WP:2637 | Interleukin-1 (IL-1) structural pathway | 0.00346 | [IL1R1, MAP2K6, MYC] |
| WP:286 | IL-3 signaling pathway | 0.00001 | [BCL2, CCR3, CRKL, RAPGEF1, STAT5A] |
| WP:2865 | IL1 and megakaryocytes in obesity | 0.00097 | [CCR3, IL1R1, PIK3CA] |
| WP:304 | Kit receptor signaling pathway | 0.00340 | [BCL2, CRKL, STAT5A] |
| WP:306 | Focal adhesion | 0.00000 | [BCL2, BIRC2, BUB1B-PAK6, CRKL, DOCK1, ITGB6, LAMA3, PAK6, PIK3CA, RAPGEF1, RASGRF1, THBS2] |
| WP:313 | Hepatocyte growth factor receptor signaling | 0.00006 | [CRKL, DOCK1, PIK3CA, RAPGEF1] |
| WP:3303 | RAC1/PAK1/p38/MMP2 pathway | 0.00192 | [MYC, PIK3CA, STAT5A] |
| WP:3651 | Pathways affected in adenoid cystic carcinoma | 0.00281 | [ARID1A, MYC, PIK3CA] |
| WP:400 | p38 MAPK signaling pathway | 0.00202 | [MAP2K6, MYC, RASGRF1] |
| WP:4141 | PI3K/AKT/mTOR - VitD3 signaling | 0.00073 | [MYC, PIK3CA, VDR] |
| WP:4205 | MET in type 1 papillary renal cell carcinoma | 0.00000 | [BUB1B-PAK6, CRKL, PAK6, PIK3CA, RAPGEF1, TFE3] |
| WP:45 | G1 to S cell cycle control | 0.00308 | [CDC25A, MYC, ORC1] |
| WP:4534 | Mechanoregulation and pathology of YAP/TAZ via Hippo and non-Hippo mechanisms | 0.00351 | [BUB1B-PAK6, ITGB6, PAK6] |
| WP:4564 | Neural crest cell migration during development | 0.00273 | [BUB1B-PAK6, PAK6, PIK3CA] |
| WP:4565 | Neural crest cell migration in cancer | 0.00321 | [BUB1B-PAK6, PAK6, PIK3CA] |
| WP:4658 | Small cell lung cancer | 0.00001 | [BCL2, BIRC2, COL4A5, LAMA3, MYC, PIK3CA] |
| WP:4698 | Vitamin D-sensitive calcium signaling in depression | 0.00273 | [BCL2, CACNA1C, VDR] |
| WP:4758 | Nephrotic syndrome | 0.00325 | [COL4A5, PAX2, ZMPSTE24] |
| WP:4816 | TGF-beta receptor signaling in skeletal dysplasias | 0.00058 | [FBN1, ITGB6, SMAD6, TFE3] |
| WP:49 | IL-2 signaling pathway | 0.00014 | [BCL2, CRKL, MYC, STAT5A] |
| WP:4906 | 3q29 copy number variation syndrome | 0.00134 | [HIF1A, MYC, STAT5A] |
| WP:560 | TGF-beta receptor signaling | 0.00346 | [ITGB6, SMAD6, TFE3] |
| WP:585 | Interferon type I signaling pathways | 0.00037 | [CRKL, MAP2K6, RAPGEF1, STAT5A] |
| WP:673 | ErbB signaling pathway | 0.00000 | [BUB1B-PAK6, CRKL, MYC, PAK6, PIK3CA, STAT5A] |
| WP:707 | DNA damage response | 0.00192 | [CDC25A, MRE11, MYC] |
| WP:712 | Estrogen signaling pathway | 0.00077 | [BCL2, GNAS, PIK3CA] |
| WP:722 | Serotonin HTR1 group and FOS pathway | 0.00202 | [MAP2K6, PIK3CA, RASGRF1] |

**Table S7.** Significant correlation between serum piRNA expressions and clinicopathological characteristics of WT patients.

| **Clinicopathological features** | **piRRNA** | **Change** | **Correlation** | **p-value** |
| --- | --- | --- | --- | --- |
| Initial lung metastasis | piR-hsa-28190 | up | -0.393397896 | 0.042344 |
| Laterality  (bilateral WT) | piR-hsa-28849 | down | 0.582581557 | 0.00143 |
|  | piR-hsa-28848 | down | 0.582581557 | 0.00143 |
|  | piR-hsa-28318 | down | 0.39079163 | 0.043854 |
| Anaplasia | piR-hsa-1913711 | down | 0.437771083 | 0.022388 |

**Table S8.** The ROC analysis of significantly differentially expressed piRNAs in WT compared to healthy controls.

| **The highest AUC values of**  **downregulated piRNAs in WT** | | | **The highest AUC values of**  **upregulated piRNAs in WT** | | |
| --- | --- | --- | --- | --- | --- |
| **piRBase Id** | **AUC** | **p-value** | **piRBase Id** | **AUC** | **p-value** |
| piR-hsa-1913711 | 0.9481 | 0.0000377 | piR-hsa-28190 | 0.9296 | 0.0000781 |
| piR-hsa-163380 | 0.8704 | 0.000667 | piR-hsa-26508 | 0.8704 | 0.000667 |
| piR-hsa-26872 | 0.8407 | 0.000168 | piR-hsa-27621 | 0.8444 | 0.001559 |
| piR-hsa-28318 | 0.8296 | 0.000975 | piR-hsa-115220 | 0.8222 | 0.002996 |
| piR-hsa-28319 | 0.8222 | 0.001268 | piR-hsa-27620 | 0.800 | 0.005904 |
| piR-hsa-28849 | 0.800 | 0.000933 | piR-hsa-27622 | 0.800 | 0.005904 |
| piR-hsa-993 | 0.7759 | 0.008306 | piR-hsa-27623 | 0.800 | 0.005904 |
| piR-hsa-24775 | 0.7667 | 0.009348 | piR-hsa-1243 | 0.7852 | 0.00889 |
| piR-hsa-1361 | 0.7481 | 0.003638 | piR-hsa-57648 | 0.7741 | 0.011351 |
| piR-hsa-3178 | 0.7481 | 0.006251 | piR-hsa-727554 | 0.7481 | 0.022951 |
| piR-hsa-28848 | 0.7333 | 0.00818 | piR-hsa-128116 | 0.7407 | 0.027358 |
| piR-hsa-148659 | 0.7259 | 0.038495 | piR-hsa-1242 | 0.7148 | 0.049232 |
| piR-hsa-12206 | 0.7259 | 0.015265 |  |  |  |
| piR-hsa-24360 | 0.7148 | 0.046304 |  |  |  |

**Supplementary Figures**


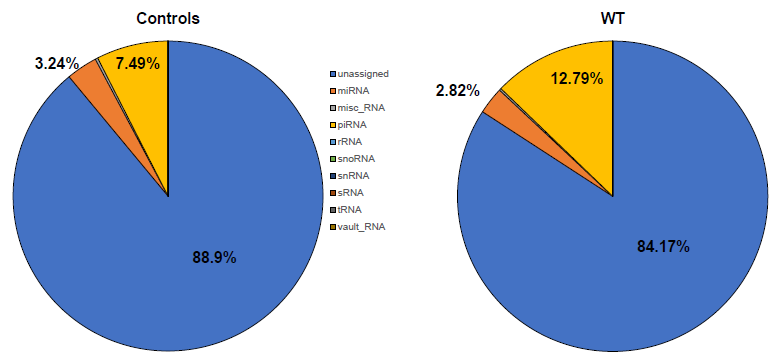


**Figure S1.** Percentages of aligned reads to different small ncRNAs in both WT and healthy control samples in our previously published study [33].


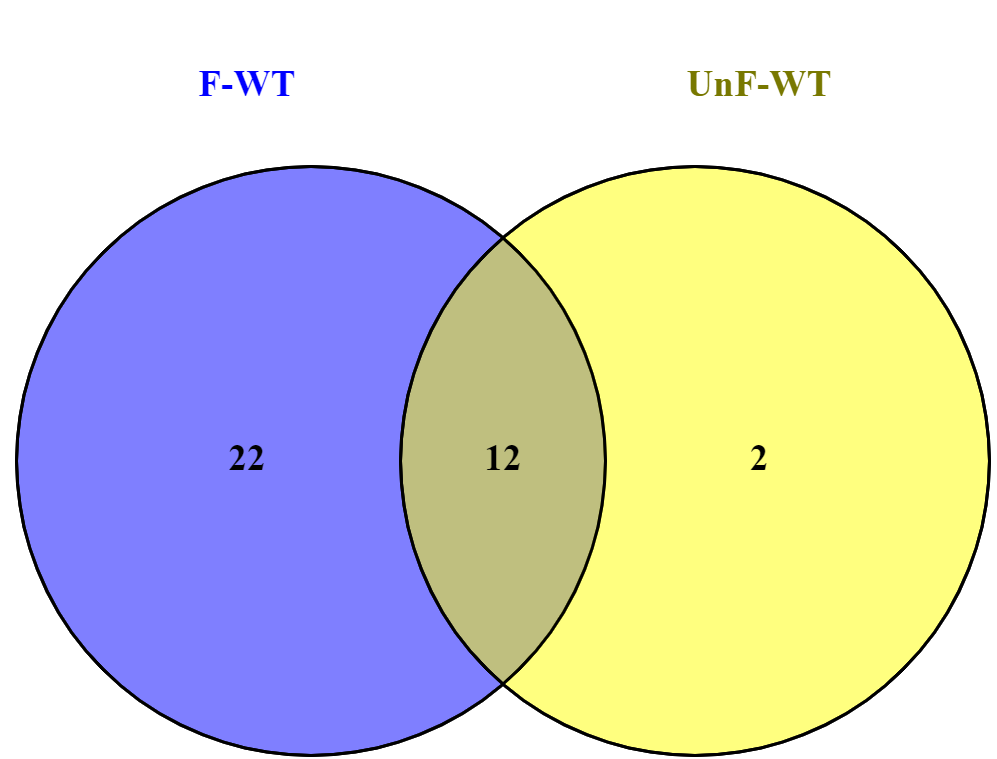


**Figure S2.** A venn diagram of the common and unique dysregulated piRNAs between Favorable and Unfavorable histology-WTs. The diagram was visualized using Venny tool v 2.1.
